# Genome of the Single Human Chromosome 18 as a “Gold Standard” for Its Transcriptome

**DOI:** 10.3389/fgene.2021.674534

**Published:** 2021-06-14

**Authors:** Ekaterina Ilgisonis, Nikita Vavilov, Elena Ponomarenko, Andrey Lisitsa, Ekaterina Poverennaya, Victor Zgoda, Sergey Radko, Alexander Archakov

**Affiliations:** Institute of Biomedical Chemistry, Moscow, Russia

**Keywords:** proteomics, transcriptomics, threshold, human genome, qPCR, Illumina RNASeq, Oxford Nanopore Technologies MinION

## Abstract

The cutoff level applied in sequencing analysis varies according to the sequencing technology, sample type, and study purpose, which can largely affect the coverage and reliability of the data obtained. In this study, we aimed to determine the optimal combination of parameters for reliable RNA transcriptome data analysis. Toward this end, we compared the results obtained from different transcriptome analysis platforms (quantitative polymerase chain reaction, Illumina RNASeq, and Oxford Nanopore Technologies MinION) for the transcriptome encoded by human chromosome 18 (Chr 18) using the same sample types (HepG2 cells and liver tissue). A total of 275 protein-coding genes encoded by Chr 18 was taken as the gene set for evaluation. The combination of Illumina RNASeq and MinION nanopore technologies enabled the detection of at least one transcript for each protein-coding gene encoded by Chr 18. This combination also reduced the probability of false-positive detection of low-copy transcripts due to the simultaneous confirmation of the presence of a transcript by the two fundamentally different technologies: short reads essential for reliable detection (Illumina RNASeq) and long-read sequencing data (MinION). The combination of these technologies achieved complete coverage of all 275 protein-coding genes on Chr 18, identifying transcripts with non-zero expression levels. This approach can improve distinguishing the biological and technical reasons for the absence of mRNA detection for a given gene in transcriptomics.

## Introduction

One of the key steps in transcriptome profiling is to determine the criteria for uncovering gene expression; that is, to establish the appropriate threshold for identifying whether or not a gene is expressed. Despite the widespread use of sequencing methods, it is commonly recognized that the choice of threshold (i.e., the cutoff level after which the signal is considered reliable) depends on the specific task being solved, sample type, and technology used (Sha et al., 2015). In particular, different sequencing technologies use different units to measure expression levels, such as reads per kilobase per million (RPKM), transcripts per kilobase per million, fragments per kilobase million, copies per cell, or number of cycles (Bullard et al., 2010).

Regardless of the chosen measurement unit, there is a tendency for an increase in the cutoff level to cause a decrease in the number of registered transcripts, thereby increasing the reliability of detection (Łabaj and Kreil, 2016; Zhao et al., 2020). This tendency has also been confirmed in targeted polymerase chain reaction (PCR)-based transcriptome mining, in which increasing the number of cycles in droplet digital PCR transcriptome profiling confirmed the presence of transcripts that scored below the cutoff level in the sample (Radko et al., 2019).

However, there is a need for a “gold standard” transcriptome data analysis, which would enable obtaining complete transcriptome coverage of the genome of interest, such as that encoded by a single chromosome. In this study, we sought to establish such a gold standard using human chromosome 18 (Chr 18) as an example. We performed comparative analyses of sequencing from previously published transcriptome datasets (Zgoda et al., 2013; Ponomarenko et al., 2014; Poverennaya et al., 2016; Radko et al., 2019)obtained with three different methods applied to the same sample of biological materials: quantitative PCR (qPCR), Illumina RNASeq (Illumina), and the recently developed nanopore sequencing platform MinION developed by Oxford Nanopore Technologies (ONT) (Jain et al., 2016). ONT can produce long reads of more than10^4^ nucleotides, which is an advantage compared with the Illumina platform that produces reads for sequences up to 300 nucleotides in length (Slatko et al., 2018). The disadvantage of ONT is that long reads contain errors at a rate of approximately one lost or misread site per 100 sequenced nucleotides (Amarasinghe et al., 2020). At present, ONT is the only sequencing technology that offers real-time analysis (for rapid insights) in fully scalable formats from the pocket to population scale, which can enable analyses of native DNA or RNA, and can sequence fragments of any length to achieve short to ultra-long read lengths. Transcript sets encoded by 275 protein-coding genes on Chr 18 measured using these three independent approaches (qPCR, Illumina, and ONT) in the HepG2 cell line and human liver tissue samples were used for this comparative analysis.

The aim of this study was to establish the optimal technology or combination of technologies for transcriptome analysis based on obtaining the maximum number of detected products at the mRNA level along with complete transcriptome coverage depending on the selected cutoff level for each platform. It is presumed that the lowest possible cutoff level leads to maximum coverage because of the reduction in unreliable results. The confirmation of low-copy transcripts with the three different technologies could therefore be used to judge the reliability of the results obtained. These results can be applied to establishing gold standard approaches for transcriptome analyses of other human chromosomes in the future.

## Materials and Methods

### Data

The results of transcriptome profiling using three technologies (qPCR, RNASeq, and ONT) of Chr 18 genes in the liver tissue and HepG2 cell line obtained by Russian Consortium were analyzed. The details of the samples, sample preparation, and experimental procedures are described in Krasnov et al. (2020). It is necessary to specify, that our study deals only with RNA transcriptome data. Datasets were previously published in the Russian Proteomic Consortium annual reports (Ponomarenko et al., 2014; Poverennaya et al., 2016; Archakov et al., 2019).

### Tanimoto Index

Bajusz et al. (2015) demonstrated that the Tanimoto index (Rogers and Tanimoto, 1960) is one of the best measures for assessing similarity, and is now widely used in chemoinformatics and bioinformatics. In particular, they ranked the performances and correlations of eight similarity metrics, which were statistically analyzed using the sum of ranking differences and analysis of variance. They found that the Cosine, Dice, Tanimoto, and Soergel similarity metrics had equivalent high performance, whereas the similarity measures derived from Euclidean and Manhattan distances were far from optimal. Based on this finding, we used the Tanimoto index to estimate the similarity among the results of transcriptomic profiling using the three different technologies.

Specifically, the coefficient of semantic similarity T (**a**, **b**) between two objects **a** and **b** is calculated using the Tanimoto normalization equation (Rogers and Tanimoto, 1960):

(1) $$T\,\left(a,b\right)=\frac{\left|P_{\text{ab}}\right|}{\left|P_{a}\right|+\left|P_{b}\right|-\left|P_{\text{ab}}\right|}$$

where *Pa* indicates the variety of transcripts *a*, *Pb* indicates the variety of transcripts *b*, and *Pab* indicates the variety of transcripts shared in *a* and *b*.

If the Tanimoto index is within 1.0–0.7, it is considered that the two sets are identical, Tanimoto index values from 0.75 to 0.55 indicate that the similarity is much weaker, and values of 0.55 and below indicate that the arrays differ considerably.

### Cutoff Level

There is currently no standard guideline for defining the low expression or noise threshold in transcriptomics; therefore, the researchers suggest the approach to determining a threshold for expression above noise: to compare the number of genes expressed at different cutoffs across all samples (Koch et al., 2018). In this work, we used cutoff levels that have been generally recommended in the related literature and compared the number of transcripts obtained depending on the cutoff level. In particular, we applied the following cutoff levels for comparison: 0 (Dall’Agnol et al., 2014), 0.1 (Abdullah et al., 2016), 1 (Xu et al., 2016; Łabaj and Kreil, 2016), 5 (Yang and Chen, 2019), and 10(Wright et al., 2013).

This approach takes into account a variety of factors, including the sequencing depth, batch effects, and technical variability. The resulting cutoff value will not only impact the number of genes to be trimmed from the original dataset but may also affect the interpretation of individual gene expression graphs.

### Reliability of the Results

In our work, we proceed from considerations that the more technologies a transcript has been detected, the more reliable its detection is. If a transcript is detected by only one technology, we do not know if this is due to the peculiarities of a particular sequencing technology or a false-positive result. At least two reasons can lead to false positive results. First, the presence of DNA in the RNA preparation. The second is the erroneous mapping of readings to genes due to the read length or the high error rate. Within the framework of this study, we cannot accurately determine the reason for the occurrence of unreliable results, since the main purpose of this study is to compare the results obtained by various technological platforms. Moreover, the lower the abundance of transcript, the less reliable the result is usually considered to be.

## Results and Discussion

**FIGURE 1:**
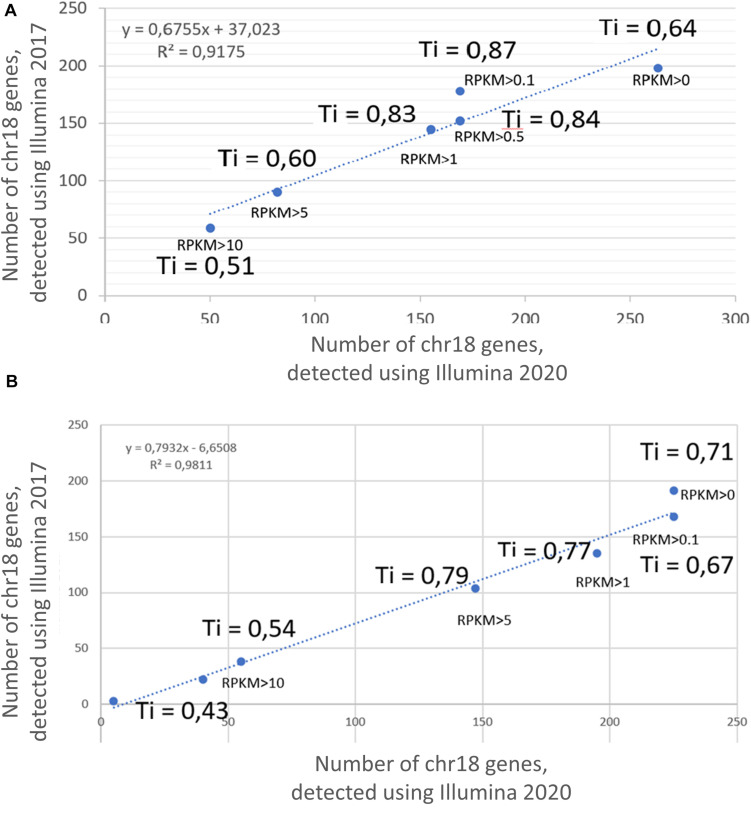
Correlation between the results of transcriptome profiling of the **(A)** HepG2 cell line and **(B)** liver, using the Illumina platform in 2017 and 2020. X axis corresponds to the number of genes, detected using Illumina 2020; Y axis corresponds to the number of genes, detected using Illumina 2017. Ti, Tanimoto index.

Transcriptomic profiling using the Illumina platform (RNASeq) was reported in two different studies by Poverennaya et al. (2017) and by Vavilov et al. (2020). Figure 1 shows the results obtained in 2017 and 2020 at different RPKM levels, demonstrating 90% correspondence; therefore, only the results obtained in 2020 were used for the comparative analysis among the three technologies in this study.

The Tanimoto index showed a tendency to increase with an increase in the cutoff level, which was a consistent trend for both the HepG2 cell line (Figure 1A) and in the liver tissue (Figure 1B). The greatest similarity between the transcripts obtained in 2017 and 2020 was found at cutoff levels of >0.1, >0.5, and >1 for the HepG2 cell line and >0.1, >5, and >5 for liver tissue. In addition, the qualitative composition of the transcripts detected by the Illumina platform in 2017 and 2020 at different cutoff levels did not differ significantly, especially observed at RPKM cutoff levels of 0, 0.1, and 1. However, the composition of the arrays at an RPKM cutoff level of >5 differed significantly between years both in the HepG2 cell line and in the liver tissue (Tanimoto index of 0.51 and 0.43, respectively). This discrepancy between the arrays is most likely due to the lifespan of the transcripts and that highly abundant transcripts disintegrate faster, which would lead to differences in transcript detection when samples are analyzed 3 years apart.

**FIGURE 2:**
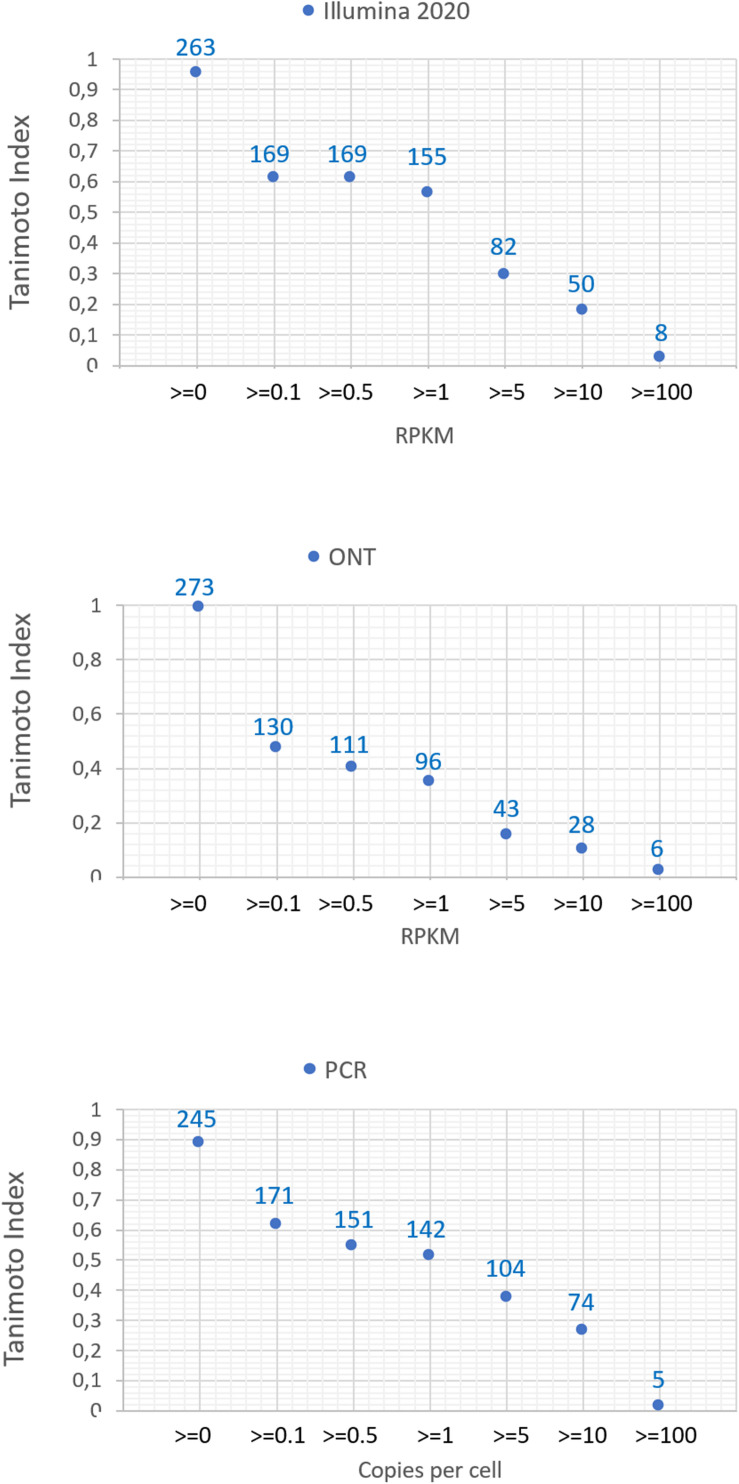
Dependence of the number of detected transcripts of chromosome 18 for various platforms (Illumina 2020 data, ONT, and qPCR) on the cutoff level and the concordance of the results obtained with the known genome of chromosome 18.

The number of common transcripts detected by the different technologies varied depending on the cutoff level. Figure 2 shows that the largest number of detected transcripts corresponded to a cutoff level of >0. With an increase in the cutoff level to 0.1, the number of detected transcripts dropped sharply. This may be attributed to noise pollution of the signal in the range from 0 to 0.1.

Regardless of the specific technology used, employing the cutoff level of 0.1 led to a decrease in the number of detected transcripts by 40–60%, and a cutoff level of 0.1 and above led to a decrease in the detected transcripts by 40–50%. The Tanimoto index decreased to 0.6, and then further decreased to 0 at higher cutoffs, indicating that transcripts for most of genes of Chr18 remained unrecorded. This may be due to the contamination of DNA in the RNA preparation or the erroneous mapping of readings to genes. To reveal the most reliable results, the intersection of sets of transcripts obtained by the three different technologies (Illumina, ONT, and qPCR) in the HepG2 cell line and in the liver tissue were compared.

**FIGURE 3:**
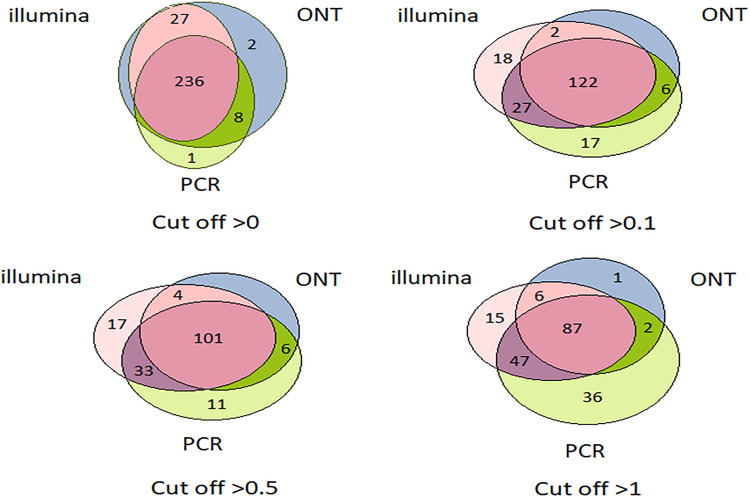
Venn diagrams showing the detection of HepG2 cell line transcripts by the Illumina, ONT, and qPCR platforms depending on the selected cutoff level.

**FIGURE 4:**
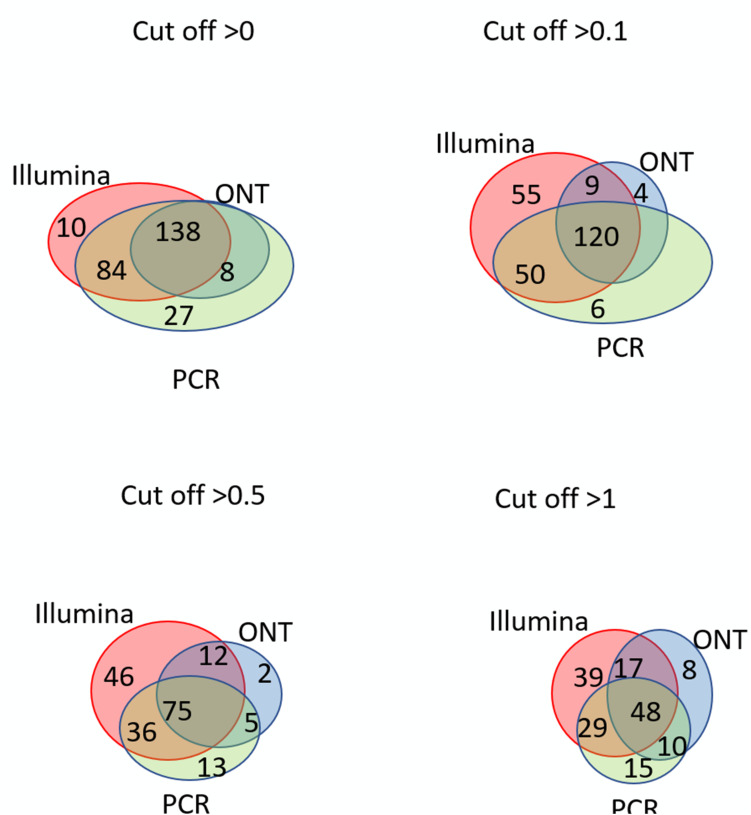
Venn diagrams showing the detection of chromosome 18 transcripts in the liver by the Illumina, ONT, and qPCR platforms depending on the selected cutoff level.

Venn diagrams representing the number of intersecting (common) transcripts according to different cutoff levels for different technologies in the HepG2 cell line and liver tissue are shown in Figure 3 and Figure 4, respectively. In HepG2 cells, a total of 236 transcripts were common to all three technologies, whereas 138 transcripts in the liver tissue were commonly identified; however, the total number of registered transcripts was 273 and 267, respectively. With an increase in the cutoff level to 0.1 and higher, the number of common transcripts obtained with the three platforms decreased sharply, whereas the number of transcripts detected by each platform increased. This increase in the number of intersecting transcripts with a decrease in the cutoff level reflects an increase in the sensitivity of each technology, making it possible to exclude the significant role of unreliable results in the expression of the Chr 18 genome (RPKM > 0), despite the theoretical existence of such a possibility.

The intersection of the results obtained by the three technologies was maximal at the minimum cutoff level (>0) for both the liver and HepG2 cells (Figures 3, 4). Importantly, this shows that applying the same minimal cutoff with different technologies will reveal the same reliable transcripts.

Interestingly, at different cutoff levels, the different technologies showed different patterns of increase in specific transcripts that were detected with only one technology. The maximum increase in the number of transcripts detected by a single technology in the HepG2 cell line was 36, which was obtained using qPCR at a cutoff level ≥1, and was 55 using Illumina in the liver tissue. Therefore, different transcripts are detected by different platforms according to variation in sensitivities, highlighting the importance of using several technologies to obtain a reliable transcriptome.

Figures 3, 4 further show that an increase in the cutoff level leads to a decrease in the total portion of transcripts detected by the three technologies. In the HepG2 cell line, at a cutoff level >0, over 236 transcripts were obtained by the three platforms, which represents more than 50% of the Chr 18 genome, and at a cutoff level >1, the number of common transcripts sharply dropped to 48, representing only 20% of the chromosome genome. The same trend was found for the liver tissue.

**FIGURE 5:**
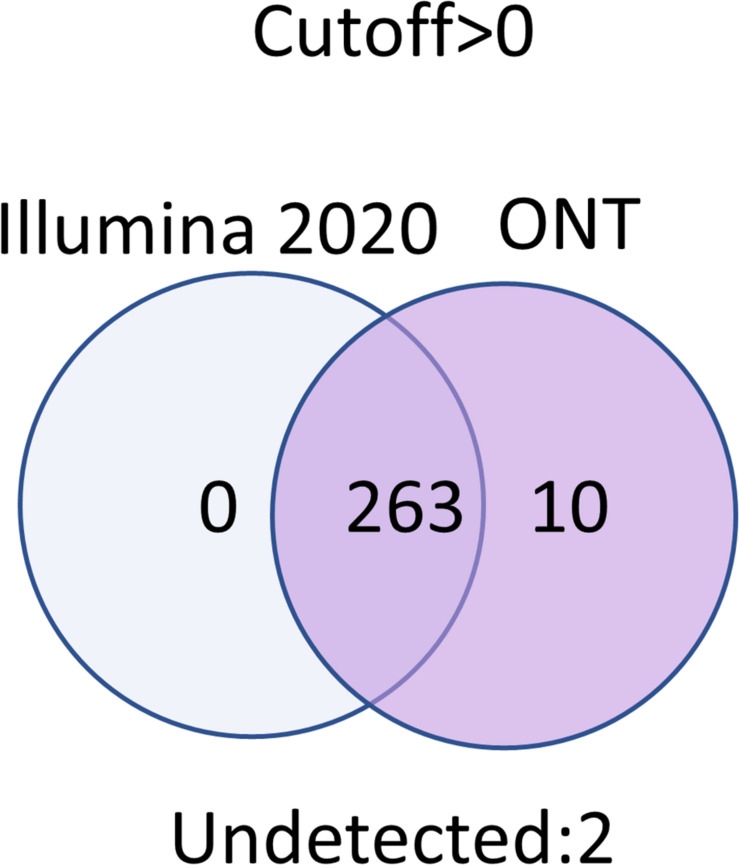
Results obtained using the Illumina and ONT technologies at a cutoff level >0.

Transcripts that were not detected by any technology at any cutoff level corresponded to two proteins: Q6ZTR6 and Q9HC47. According to the UniProt database (accession date—02.2021) (Apweiler et al., 2004), these proteins also could not be confirmed (Figure 5). Q6ZTR6 is annotated as a “predicted” protein, and Q9HC47 corresponds to cutaneous T-cell lymphoma-associated antigen 1 protein, which is annotated at a PE2 level (protein evidence confirmed at the transcript level). These findings suggested that these missing transcripts did not actually correspond to missing protein detection on these platforms. Ten transcripts were obtained using only ONT technology, which could be considered false positives (Figure 5). To assess this possibility, we screened the complete genomes of the liver and HepG2 cell lines obtained from an RNASeq database (accession date—02.2021) (Edgar et al., 2002), demonstrating that these unique transcripts found using ONT technology have no homologous sequence to genes on any other chromosomes besides Chr 18. This suggested that these undetected transcripts are likely the result of extremely underrepresented gene expression on Chr 18 (Supplementary Material). Of course, detection of these transcripts could be a results of DNA contamination or wrong mapping of poor quality nanopore reads, but we cannot estimate it in the course of this research.

Thus, the use of two technologies, Illumina and ONT, enabled the identification of transcripts corresponding to all experimentally observed proteins derived from genes located on human Chr 18, with the exception of two transcripts that were also not confirmed at the protein level in the Nextprot database (accession date—02.2021) (Zahn-Zabal et al., 2020).

## Conclusion

The greatest coverage of the human genome encoded by Chr 18 was achieved at a cutoff level of >0. Among the three technologies compared (qPCR, Illumina, and ONT), Illumina sequencing and nanopore technology (ONT) complement each other well in terms of non-overlapping common transcripts and detection the complete set of protein-coding genes encoded by the chromosome. In particular, the combined use of Illumina RNASeq and ONT revealed 98–100% of transcripts of the Chr 18 genome at a cutoff level of 0. We also found an expected result that the lowest possible cutoff level leads to maximum coverage due to the lack of unreliable results. However, confirmation of the existence of low-copy transcripts when using all three technologies could further ensure the reliability of the results obtained. This was evidenced by the comparison of the Tanimoto index, which decreased with an increasing cutoff level (Figure 2). At a cutoff level of 0.1 and higher, the Tanimoto index was reduced to 0.6 or less, which indicates that under these conditions, the transcriptome obtained would differ significantly from the full Chr 18 exome.

## Data Availability Statement

The original contributions presented in the study are included in the article/Supplementary Material, further inquiries can be directed to the corresponding author/s.

## Author Contributions

EI: manuscript draft. NV: analysis and interpretation of data. ElP: project administration. AL: critical revision. EkP: analysis and interpretation of data. VZ: critical revision. SR: acquisition of data. AA: study conception and design. All authors contributed to the article and approved the submitted version.

## Conflict of Interest

The authors declare that the research was conducted in the absence of any commercial or financial relationships that could be construed as a potential conflict of interest.

## References

[B1] Abdullah H. M. Akbari P. Paulose B. Schnell D. Qi W. Park Y. (2016). Transcriptome profiling of *Camelina sativa* to identify genes involved in triacylglycerol biosynthesis and accumulation in the developing seeds. *Biotechnol. Biofuels* 9:136. 10.1186/s13068-016-0555-5 27382413 PMC4932711

[B2] Amarasinghe S. L. Su S. Dong X. Zappia L. Ritchie M. E. Gouil Q. (2020). Opportunities and challenges in long-read sequencing data analysis. *Genome Biol.* 21:30. 10.1186/s13059-020-1935-5 32033565 PMC7006217

[B3] Apweiler R. Bairoch A. Wu C. H. Barker W. C. Boeckmann B. Ferro S. (2004). UniProt: the universal protein knowledgebase. *Nucleic Acids Res.* 32 D115–D119. 10.1093/nar/gkh131 14681372 PMC308865

[B4] Archakov A. I. Aseev A. L. Bykov V. A. Grigoriev A. I. Govorun V. M. Ilgisonis E. V. (2019). Challenges of the human proteome project: 10-year experience of the Russian Consortium. *J. Proteome Res.* 18 4206–4214. 10.1021/acs.jproteome.9b00358 31599598

[B5] Bajusz D. Rácz A. Héberger K. (2015). Why Is Tanimoto index an appropriate choice for fingerprint-based similarity calculations? *J. Cheminform.* 7:20. 10.1186/s13321-015-0069-3 26052348 PMC4456712

[B6] Bullard J. H. Purdom E. Hansen K. D. Dudoit S. (2010). Evaluation of statistical methods for normalization and differential expression in MRNA-seq experiments. *BMC Bioinformatics* 11:94. 10.1186/1471-2105-11-94 20167110 PMC2838869

[B7] Dall’Agnol H. P. M. B. Baraúna R. A. de P. H. C. G. Sá R. T.J. Ramos Nóbrega F. Nunes C. I. P. (2014). Omics profiles used to evaluate the gene expression of *Exiguobacterium antarcticum* B7 during cold adaptation. *BMC Genomics* 15:986. 10.1186/1471-2164-15-986 25407400 PMC4247613

[B8] Edgar R. Domrachev M. Lash A. E. (2002). Gene expression omnibus: ncbi gene expression and hybridization array data repository. *Nucleic Acids Res.* 30 207–210. 10.1093/nar/30.1.207 11752295 PMC99122

[B9] Jain M. Olsen H. E. Paten B. Akeson M. (2016). The Oxford Nanopore MinION: delivery of nanopore sequencing to the genomics community. *Genome Biol.* 17 1–11. 10.1186/s13059-016-1103-0 27887629 PMC5124260

[B10] Koch C. M. Chiu S. F. Akbarpour M. Bharat A. Ridge K. M. Bartom E. T. (2018). A beginner’s guide to analysis of RNA sequencing data. *Am. J. Respir. Cell Mol. Biol.* 59 145–157. 10.1165/rcmb.2017-0430TR 29624415 PMC6096346

[B11] Krasnov G. S. Radko S. P. Ptitsyn K. G. Shapovalova V. V. Timoshenko O. S. Khmeleva S. A. (2020). Human Chr18: ‘Stakhanovite’ genes, missing and UPE1 proteins in liver tissue and HepG2 cells. *BioRxiv* [Preprint]. 10.1101/2020.11.04.358739

[B12] Łabaj P. P. Kreil D. P. (2016). Sensitivity, specificity, and reproducibility of RNA-seq differential expression calls. *Biol. Direct* 11:66. 10.1186/s13062-016-0169-7 27993156 PMC5168849

[B13] Ponomarenko E. A. Kopylov A. T. Lisitsa A. V. Radko S. P. Kiseleva Y. Y. Kurbatov L. K. (2014). Chromosome 18 transcriptoproteome of liver tissue and HepG2 cells and targeted proteome mapping in depleted plasma: update 2013. *J. Proteome Res.* 13 183–190. 10.1021/pr400883x 24328317

[B14] Poverennaya E. V. Ilgisonis E. V. Ponomarenko E. A. Kopylov A. T. Zgoda V. G. Radko S. P. (2017). Why are the correlations between MRNA and protein levels so low among the 275 predicted protein-coding genes on human chromosome 18. *J. Proteome Res.* 16 4311–4318. 10.1021/acs.jproteome.7b00348 28956606

[B15] Poverennaya E. V. Kopylov A. T. Ponomarenko E. A. Ilgisonis E. V. Zgoda V. G. Tikhonova O. V. (2016). State of the art of chromosome 18-Centric HPP in 2016: transcriptome and proteome profiling of liver tissue and HepG2 cells. *J. Proteome Res.* 15 4030–4038. 10.1021/acs.jproteome.6b00380 27527821

[B16] Radko S. P. Poverennaya E. V. Kurbatov L. K. Ponomarenko E. A. Lisitsa A. V. Archakov A. I. (2019). The ‘Missing’ proteome: undetected proteins, not-translated transcripts, and untranscribed genes. *J. Proteome Res.* 18 4273–4276. 10.1021/acs.jproteome.9b00383 31621326

[B17] Rogers D. J. Tanimoto T. T. (1960). A Computer program for classifying plants. *Science* 132 1115–1118. 17790723 10.1126/science.132.3434.1115

[B18] Sha Y. Phan J. H. Wang M. D. (2015). “Effect of low-expression gene filtering on detection of differentially expressed genes in RNA-seq data,” in *Proceedings of the Annual International Conference of the IEEE Engineering in Medicine and Biology Society, EMBS, 2015-Novem:6461–64* (New York, NY: Institute of Electrical and Electronics Engineers Inc), 10.1109/EMBC.2015.7319872 PMC4983442 26737772

[B19] Slatko B. E. Gardner A. F. Ausubel F. M. (2018). Overview of next generation sequencing technologies (and bioinformatics) in cancer. *Curr. Protoc. Mol. Biol.* 122:e59. 10.1002/cpmb.59 29851291 PMC6020069

[B20] Vavilov N. E. Zgoda V. G. Tikhonova O. V. Farafonova T. E. Shushkova N. A. Novikova S. E. (2020). Proteomic analysis of Chr 18 proteins using 2D fractionation. *J. Proteome Res.* 19 4901–4906. 10.1021/acs.jproteome.0c00856 33202127

[B21] Wright H. L. Thomas H. B. Moots R. J. Edwards S. W. (2013). RNA-seq reveals activation of both common and cytokine-specific pathways following neutrophil priming. *PLoS One* 8:e58598. 10.1371/journal.pone.0058598 23554905 PMC3590155

[B22] Xu J. Gong B. Wu L. Thakkar S. Hong H. Tong W. (2016). Comprehensive assessments of RNA-seq by the SEQC consortium: FDA-led efforts advance precision medicine. *Pharmaceutics* 8:8. 10.3390/pharmaceutics8010008 26999190 PMC4810084

[B23] Yang J. R. Chen X. (2019). Dosage sensitivity of X-linked genes in human embryonic single cells. *BMC Genomics* 20:42. 10.1186/s12864-019-5432-8 30642250 PMC6332578

[B24] Zahn-Zabal M. Michel P. A. Gateau A. Nikitin F. Schaeffer M. Audot E. (2020). The neXtProt knowledgebase in 2020: data, tools and usability improvements. *Nucleic Acids Res.* 48 D328–D334. 10.1093/nar/gkz995 31724716 PMC7145669

[B25] Zgoda V. G. Kopylov A. T. Tikhonova O. V. Moisa A. A. Pyndyk N. V. Farafonova T. E. (2013). Chromosome 18 transcriptome profiling and targeted proteome mapping in depleted plasma, liver tissue and HepG2 cells. *J. Proteome Res.* 12 123–134. 10.1021/pr300821n 23256950

[B26] Zhao S. Ye Z. Stanton R. (2020). Misuse of RPKM or TPM normalization when comparing across samples and sequencing protocols. *RNA* 26 903–909. 10.1261/RNA.074922.120 32284352 PMC7373998

